# Supplementary material for: Relationship between lymphedema after breast cancer treatment and biophysical characteristics of the affected tissue
Source: PLoS One. 2022 Apr 20;17(4):e0264160. doi: 10.1371/journal.pone.0264160 (PMC9020674; doi:10.1371/journal.pone.0264160)
Supplement: Attachment — Submitted filename: Response.docx [file pone.0264160.s001.docx]

**Ms. Ref. No.: PONE-D-20-18187 – Title: Relationship between lymphedema after breast cancer treatment and biophysical characteristics of the affected tissue**

**PLOS ONE**

Thank you for your valuable inputs towards our study. The text described below respond to the editor. They emphasize the relevant data and enhance comprehension of the manuscript.

**Comments to the Author:** There are already published several articles in literature with the same topic about lymphedema and breast cancer. There is no new content and the form is not suitable for PlosOne journal publication.

**Answer:** The thematic evaluation of lymphedema and characterization of intrinsic tissue changes is vast in the literature because it is such an incident and complex diagnosis. Faced with different perspectives for the evaluation of lymphedema, the submitted manuscript brings a new perception for evaluation.

In highlight, it is noted that the submitted study has as sub-theme the evaluation through diagnostic ultrasound, a tool that was able to see, evaluate, and quantify the subcutaneous tissue affected by lymphedema. A rigorous evaluation protocol was used, a phantom was developed to control the variables, and additional plugins to software for quantification, this study is innovative in characterizing and quantifying measures of image texture in modeB and its topography in the upper limbs.

Breast cancer is the most common malignancy in women worldwide (Ghoncheh et al., 2016). Lymphedema of the upper limbs is the most common and worrying morbidity after surgical and adjuvant treatment (De Brucker et al., 2016). Estimates of lymphedema prevalence are relatively high, one in five women who survive breast cancer may develop lymphedema at some point in their life (DiSipio et al., 2013).

Lymphedema is a chronic disease that needs control and early diagnosis (Rockson 2018). The absence of a cure for lymphedema is due, among other factors, to an incomplete understanding of the pathophysiological changes that result in lymphedema. Lymphedema can actually be diagnosed infrequently by professionals in general. This problem may be related to the professionals' unfamiliarity with the disease and the lack of non-invasive diagnostic tests that are readily available to trained professionals (Denlinger et al., 2018).

Although the recommendations of the guidelines may be typically delayed in relation to new developments in the diagnostic field, changes in the guidelines depend on the robust evidence needed for diagnostic ultrasound, this study meets this objective by bringing new protocols. Certainly, the immediate availability and the non-invasive nature of ultrasound can make this modality the first choice to establish the diagnosis of lymphedema, provided that clinical measures for this tool are well described and quantified in the literature.

References

De Brucker B ,Zeltzer A, Seidenstuecker K, Hendrickx B, Adriaenssens N, Hamdi M. Breast Cancer-Related Lymphedema: Quality of Life after Lymph Node Transfer. Plast Reconstr Surg. 2016;137(6):1673-80.

Rockson SG, Rivera KK. Estimating the population burden of lymphedema. Ann N Y Acad Sci. 2008;1131:147-54.

DiSipio T, Rye S, Newman B, Hayes S. Incidence of unilateral arm lymphoedema after breast cancer: a systematic review and meta-analysis. Lancet Oncol. 2013 May;14(6):500-15.

Rockson SG: Lymphedema after Breast Cancer Treatment. N Eng J Med. 2018; 379: 1937-1944.

Denlinger CS, Sanft T, Baker KS, Broderick G, Demark‐Wahnefried W, Friedman DL, Goldman M, Hudson M, Khakpour N, King A, et al. Survivorship, version 2.2018, NCCN clinical practice guidelines in oncology. J Natl Compr Cancer Netw . 2018; 16: 1216- 1247.
